# Aversive Pavlovian Responses Affect Human Instrumental Motor Performance

**DOI:** 10.3389/fnins.2012.00134

**Published:** 2012-10-08

**Authors:** Francesco Rigoli, Enea Francesco Pavone, Giovanni Pezzulo

**Affiliations:** ^1^Università di SienaSiena, Italy; ^2^Istituto di Scienze e Tecnologie della Cognizione, Consiglio Nazionale delle RicercheRoma, Italy; ^3^Università di Roma “La Sapienza,”Roma, Italy; ^4^Fondazione Santa Lucia Istituto Di Ricovero e Cura a Carattere ScientificoRoma, Italy; ^5^Istituto di Linguistica Computazionale “Antonio Zampolli,” Consiglio Nazionale delle RicerchePisa, Italy

**Keywords:** controllability, goal-directed, habitual, Pavlovian, reinforcement learning

## Abstract

In neuroscience and psychology, an influential perspective distinguishes between two kinds of behavioral control: instrumental (habitual and goal-directed) and Pavlovian. Understanding the instrumental-Pavlovian interaction is fundamental for the comprehension of decision-making. Animal studies (as those using the negative auto-maintenance paradigm), have demonstrated that Pavlovian mechanisms can have maladaptive effects on instrumental performance. However, evidence for a similar effect in humans is scarce. In addition, the mechanisms modulating the impact of Pavlovian responses on instrumental performance are largely unknown, both in human and non-human animals. The present paper describes a behavioral experiment investigating the effects of Pavlovian conditioned responses on performance in humans, focusing on the aversive domain. Results showed that Pavlovian responses influenced human performance, and, similar to animal studies, could have maladaptive effects. In particular, Pavlovian responses either impaired or increased performance depending on modulator variables such as threat distance, task controllability, punishment history, amount of training, and explicit punishment expectancy. Overall, these findings help elucidating the computational mechanisms underlying the instrumental-Pavlovian interaction, which might be at the base of apparently irrational phenomena in economics, social behavior, and psychopathology.

## Introduction

In psychology and neuroscience, an influential perspective (the multicontroller framework) views human and animal behavior as the result of the interaction among instrumental (goal-directed and habitual) and Pavlovian systems (Mackintosh, 1983; Balleine and Dickinson, 1998; Daw et al., 2005; Dayan and Seymour, 2008; Balleine and O’Doherty, 2009). Contrary to instrumental controllers, which learn novel actions guided by reward maximization (Daw et al., 2005; Pezzulo and Castelfranchi, 2009; Pezzulo and Rigoli, 2011; Solway and Botvinick, 2012), the Pavlovian system associates hard-wired reactions to unconditioned or conditioned stimuli (Mackintosh, 1983; Dayan and Seymour, 2008). The instrumental-Pavlovian interaction has been studied both in animals (Estes and Skinner, 1941; Rescorla and Solomon, 1967; Overmier et al., 1971; Dickinson and Pearce, 1977; Colwill and Rescorla, 1988; Holland, 2004) and, more recently, in humans (Bray et al., 2008; Talmi et al., 2008; Huys et al., 2011). The most widely used paradigms are Pavlovian-instrumental transfer (PIT) and conditioned suppression. These paradigms have shown that Pavlovian stimuli influence both choice and vigor of instrumental behavior. For instance, in the Bray et al.’s (2008) study, the presence of an appetitive Pavlovian stimulus led participants to choose items previously associated with that stimulus. The most plausible explanation of this finding is that Pavlovian stimuli biased the items’ value. From this and similar studies, it emerges that Pavlovian mechanisms influence goal values, while it remains unclear whether they can also influence the correct execution of an adaptive instrumental action. In relation to this, animal studies have demonstrated that Pavlovian responses can cause misbehavior, namely a paradoxical negative effect on animal’s performance (Breland and Breland, 1966; Morse et al., 1967; Brown and Jenkins, 1968; Williams and Williams, 1969; Mackintosh, 1983; Hershberger, 1986). For example, in the negative auto-maintenance paradigm (Williams and Williams, 1969), pigeons were trained with a light repeatedly paired with food. As a consequence of the food-light association learning, these animals exhibited a conditioned response of pecking the light when it appeared. Crucially, this response did not have any instrumental consequences in this phase. Afterward, in the test phase, the light appeared for some trials, and food was given to pigeons when they abstained from pecking the light. Surprisingly, pigeons continued to exhibit the pecking response, although they gained less reward. This result was interpreted as the activation of the innate Pavlovian response of approaching food-related stimuli, at the expense of a more efficient instrumental action. On the basis of this and similar evidence, it has been proposed that flexible instrumental responses can be activated together with rigid Pavlovian ones. In such circumstances, Pavlovian responses are adaptive when they are compatible with instrumental behavior. Alternatively, namely when they go in the opposite direction, Pavlovian responses are maladaptive (Dayan et al., 2006).

However, to date, the mechanisms underlying the Pavlovian influence on instrumental performance are largely unknown. This is particularly true for humans, in relation to whom evidence in favor of maladaptive Pavlovian effects on performance is scarce (Guitart-Masip et al., 2011).

## The Present Study

The general aim of the present study was to analyze the influence of Pavlovian responses on instrumental motor performance in humans. Linked to this, we aimed to test whether, and in which conditions, it was possible to detect a maladaptive effect. We focused on the *aversive domain*, a condition widely used in the animal literature (Morse et al., 1967).

A first specific aim of the study was to investigate the role of three variables as possible modulators of the Pavlovian influence on performance:

(1) Temporal threat distance (TTD): Contemporary animal models consider the spatial and TTDs as modulating defensive behavior (Fanselow and Lester, 1988; Blanchard and Blanchard, 1989; McNaughton and Corr, 2004). In line with these models, when a threat is close (rather than distant), the Pavlovian activation could increase and impair performance.
(2) Motivational Value (MV): This variable depends on the past punishment history associated with a context. It is plausible that the amount of past punishment influences both the Pavlovian value of associated stimuli and the value of the goal of avoiding the punishment in the future. In other words, many punishments in the past could increase Pavlovian activation, which in turn could impair performance. At the same time, many punishments in the past could increase the goal-directed motivation towards safety, improving performance.
(3) Controllability (CON): This variable corresponds to the difficulty of a task. More specifically, CON can be defined as the probability of achieving an outcome associated to a positive value through instrumental behavior (Huys and Dayan, 2009). Many studies, related to learned-helplessness, have described the effects of CON (Mineka and Hendersen, 1985; Maier and Watkins, 2005). Experimental findings suggest that CON is inversely correlated with the level of conditioned fear response. For example, rats showed a stronger fear response in front of uncontrollable shocks than in front of controllable ones (Mineka et al., 1984). In relation to the present study, it is possible that low CON increases Pavlovian activation, which in turn could impair performance.

A second specific aim of the study was to investigate whether the Pavlovian system exerts its influence on performance even without explicit threat expectancy. A similar issue has been investigated with respect to physiological Pavlovian responses, such as skin conductance. Evidence indicates that, at least after a certain amount of learning, a conditioned skin conductance response can be detected even without explicit threat expectancy (Schell et al., 1991; Lipp and Edwards, 2002). However, to date, the role of punishment expectancy as a modulator of Pavlovian influences on behavior is unknown. In line with skin conductance experiments, we hypothesized an influence of the Pavlovian system on performance even without explicit threat expectancy.

A third specific aim of the study was to investigate the differential Pavlovian impact on goal-directed and habitual controllers. Some theoretical proposals have argued that Pavlovian responses mostly influence the goal-directed system (Loewenstein and O’Donoghue, 2004), whereas others assert a greater influence on a habitual system (Holland, 2004; Dayan et al., 2006).

In the present paper, we describe a behavioral experiment in which we analyzed human performance in a sensorimotor instrumental task, with the aim of investigating the influence of Pavlovian responses on instrumental performance. In the task, we compared two different conditions, one in which a cue (CS+) signaled that a mistake was punished by the delivery of an electric shock, and one in which another cue (CS−) signaled that a mistake was not punished. We reasoned that, in the first condition, both the Pavlovian and the instrumental controllers should have been active, whereas, in the second one, only the latter should have. By comparing the two conditions, and manipulating also the putative modulator variables, the effect of Pavlovian mechanisms on instrumental performance should have emerged.

## Materials and Methods

### Participants

Thirty-eight volunteers (17 males and 21 females; mean age = 25 years, SD = 4.4) were recruited through the participant pool of the University of Rome “La Sapienza.” The study was approved by the Ethics Committee of the Institute of Cognitive Sciences and Technologies of the Italian National Research Council.

### Task description

**Figure 1:**
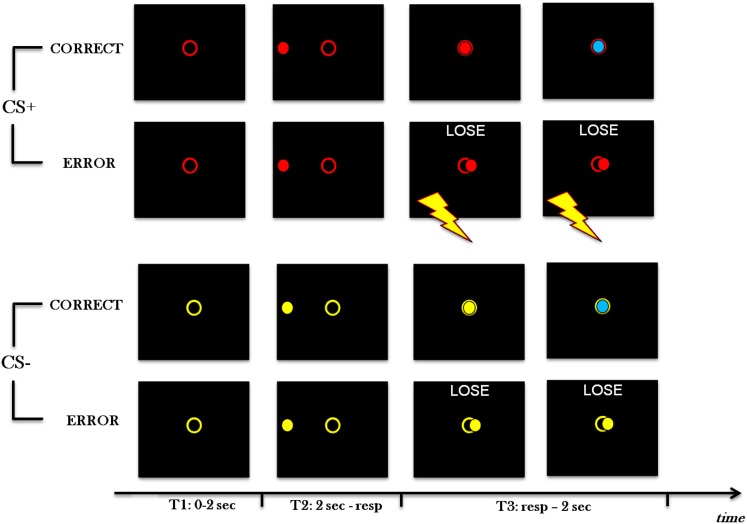
**Experimental paradigm**. Four types of trials of Block 1 and 2 are shown, in which red is CS+ and yellow is CS−. From the top, the first example is with CS+ and a correct response; the second one is with CS+ and an error; the third one is with CS− and a correct response; the fourth one is with CS− and an error. At the beginning of each trial (T1), a colored open circle is presented in the center of the screen. After 2 s (T2), a ball appears in the center of one side of the screen and moves toward the opposite side, passing through the circle. Velocity varies randomly trial-by-trial on two levels. If the participant pushes the button at the right time (T3, first and third examples), and keeps it pressed for 1 s, the ball turns blue and, after a further second, a new trial begins. If the participant presses the button too fast or too slow (T3, second and forth examples), a negative feedback is displayed for 2 s, then a new trial begins. Furthermore, in CS+ conditions (second example), immediately after a mistake, an electric shock is delivered to the index finger that is used to press, and to the medium finger of the same hand. Electric shock lasts 500 ms. Contrary to Block 1 and 2, in Block 3 shock is never delivered with CS+.

Participants sat in front of a computer black screen for a task composed of 240 trials (see Figure 1). At every trial, a colored open circle appeared in the center of the screen. The circle was red (CS+ condition) for half of the trials and yellow (CS− condition) for the other half, with random order (CS+ and CS− colors were counterbalanced across subjects). After 2 s, a ball of the same color of the circle appeared in the middle of one of the four sides of the screen and moved toward the opposite side, passing through the circle. The velocity of the ball varied randomly trial-by-trial on two levels (Fast, Slow). Participants had to press a button with the index finger of the right hand when the ball was in the circle. During both blocks 1 and 2 (80 trials each), participants either received an electric shock (in CS+ condition) or not (in CS− condition) when making an error (i.e., they either pressed too slow or too fast). The shock was delivered to the same finger the subjects used to press, that is to the index finger of the right hand, and to the medium finger of the same hand. In block 3 (80 trials), no participants received an electric shock, ever. At the beginning of block 3, participants were informed of the absence of shock delivery.

Plausibly, participants had the goal of winning at every trial. However, during trials in which mistakes were punished with shock, the goal of winning reasonably had an even stronger value. A first possibility was that performance was proportional to instrumental value, hence that it was better in CS+ than CS−. Although this hypothesis is in line with some findings (Hull, 1943; Blake et al., 2002; Pleger et al., 2008), other evidence is at odds with it (Mackintosh, 1983; Dayan and Seymour, 2008; Guitart-Masip et al., 2011). A second possibility was that, in CS+, both the instrumental and the Pavlovian systems were activated by shock threat. Instrumental and Pavlovian activation would have an opposite effect on performance, the former enhancing it, the latter impairing it.

In order to operationalize one of the putative modulator variables, namely TTD, we manipulated the ball velocity on two levels (see Discussion for the implications of this procedure). The reason why velocity and TTD are inherently associated is that, in fast trials, participants expected the threat to be close in time, and vice versa in slow trials. This was aimed at testing whether, in line with the hypothesized role of TTD, the positive instrumental effects on performance emerged in slow trials, and the negative Pavlovian effects emerged in fast trials. We hypothesized velocity to be associated also with two other putative modulator variables, namely CON (i.e., in average we expected a better performance in slow trials than fast trials) and MV (i.e., in average we expected more shocks in fast CS+ trials than slow CS+ trials). To disentangle the role of TTD, CON, and MV, we planned a between-subjects analysis and a trial-by-trial analysis, in which each variable contribution could be separated from the others.

In addition, we investigated whether the Pavlovian system exerted its influence on performance even without explicit threat expectancy. To this aim, we studied performance also in extinction, namely in a third block where shock was never delivered and participants were informed of this.

Finally, we investigated whether the Pavlovian system differentially impacted on goal-directed and habitual controllers. Although goal-directed and habitual control coexist in most contexts (Daw et al., 2005; Wunderlich et al., 2012), research has shown that the relative strength of the two systems depends on the condition. Specifically, habitual control increases with experience (Daw et al., 2005) and performance in simple motor tasks increases when habitual control grows (Doyon et al., 2003). Based on these considerations, we analyzed two blocks of trials, hypothesizing that Block 1 was mostly guided by goal-directed mechanisms, whereas Block 2 by habitual ones. In order to verify this hypothesis, we tested whether performance increased in Block 2 compared to Block 1 (see Results).

### Apparatus and materials

The experiment was conducted using E-Prime 1.2 software on a computer running Microsoft Windows. Participants sat 80 cm from a 21″ screen. Electrical pain stimulation was controlled and delivered by a Laika Excel Sport Stimulator, approved for clinical use.

### Stimuli

The screen was black. The open circle in the center of the screen had a 1.8 cm radius, the ball was a filled circle of 0.7 cm radius. The circle and the ball were either red or yellow, varying randomly across trials. The velocity of the ball varied randomly on four levels, covering 0.3 cm every 9, 10, 13, and 15 ms (corresponding to velocity 1–4, respectively). The velocity levels were chosen on the base of a preliminary investigation conducted through a pilot study. Different levels of velocity were aggregated in two groups (velocity 1 and 2 corresponded to Fast Velocity; velocity 3 and 4 corresponded to Slow Velocity).

### Procedure

Before starting the task, a silver-chloride electrode was fixed to the medium finger of participants’ right hand, while a second electrode was fixed under an aluminum layer glued upon a button. While participants repeatedly pushed and released the button with the right hand index finger, the electric stimulation was delivered and they could perceive it when they pushed. Starting from a very low level, the shock intensity was raised until each participant indicated it as quite unpleasant, just under the pain threshold. This level was adopted as punishment in the first block. The shock intensity setting procedure was repeated after the first block, and this second level was adopted in the second block. After the first and second blocks, participants were asked to evaluate the average electric stimulation received with two visual analog scales (VAS), one for intensity and one for unpleasantness.

After the first shock intensity setting, participants were fully instructed about the task. Afterward, they completed one practice block of eight trials and then three experimental blocks of 80 trials each. The practice and the three experimental blocks were all identical except that shock was not delivered in the third experimental block. At every trial, the open colored circle appeared in the center of the screen. After 2 s, the ball appeared on one side of the screen and immediately moved toward the opposite side. In order to win the trial, participants had to press the button at the right time and to keep it pressed for 1 s. Once they did it, the ball become blue and disappeared, and, after 1 s, a new trial began. If participants made a mistake, a negative feedback statement appeared for 2 s. At the same time, with the exception of the third block, in trials with CS+, an electric stimulation lasting 500 ms was delivered through the two electrodes, one fixed to the medium finger and one under the button. Following the feedback statement, a new trial started immediately. If participants did not press the button in a trial, an error feedback was presented and the trial was repeated. If participants pressed at the right time but released the button too early, an error feedback was presented. We instructed participants to keep the button pressed to avoid that they used the strategy to press and release quickly the button. This strategy could have led to a performance decrease that was not due to Pavlovian mechanisms, but to the adoption of different strategies in CS+ and CS−. In average, trials in which participants released the button were three per subject (SD = 2). In the analyses presented in this paper, these trials were scored as winnings. In order to ascertain that this scoring procedure did not affect the results, we also performed the same ANOVA analyses considering these trials as errors, obtaining equivalent results.

Before the third block, participants were informed about the absence of shock delivery, and the electrodes were removed. This procedure was taken from a previous study (Lipp and Edwards, 2002), as it revealed to be more effective in enhancing participants’ trust than instructions delivery only. Moreover, at the end of the experiment, we asked participants to rate their confidence on the absence of shock in the third block. This was done to double-check that participants fully trusted the instructions.

## Results

### Aggregated within-subjects analysis

As a first analysis, we conducted a repeated-measures analysis of variance (ANOVA) on the aggregated data with three independent variables (2 × 3 × 2): Stimulus (CS+ or CS−), Block (first, second, or third), and Velocity (Slow or Fast). We first performed this analysis using the average distance from the target-circle as dependent variable (see Table 1 for means and SD in the different conditions). Importantly, distance was scored as negative when participants pressed too early, and as positive when they pressed too late. This analysis revealed a main effect of Velocity [*F*(1,37) = 56.41, *p* = 0.000, $\eta_{p}^{2}=0.61$; in this and in all following analyses the threshold for statistical significance was set to 0.05]. In other words, participants pressed the button at a larger distance in Fast Velocity than in Slow Velocity. All other main effects were non-significant [main affect of Block: *F*(2,37) = 0.26, *p* = 0.76; main effect of Stimulus: *F*(1,37) = 0.09, *p* = 0.76]. In addition, a Block × Velocity interaction was found [*F*(2,74) = 4.4, *p* = 0.016, $\eta_{p}^{2}=0.12$]. All other interactions were non-significant [Stimulus-Block: *F*(2,74) = 2.44, *p* = 0.09; Stimulus-Velocity: *F*(1,37) = 0.2; *p* = 0.96; Velocity-Block-Stimulus: *F*(2,74) = 2.47; *p* = 0.092].

**Table 1:** **Means and SD of distance and performance relative to the experimental conditions**.

Condition	Distance		Performance	
	Mean	SD	Mean	SD
CS+, B1, F	−9.6	19.9	26.9	14.9
CS−, B1, F	−8.7	18.0	31.7	15.7
CS+, B1, S	5.1	26.7	49.7	20.4
CS−, B1, S	9.8	19.6	46.4	18.0
CS+, B2, F	−6.0	13.2	34.3	13.9
CS−, B2, F	−5.0	11.6	39.1	16.0
CS+, B2, S	6.7	15.9	60.7	17.8
CS−, B2, S	5.0	13.1	54.1	18.0
CS+, B3, F	−7.6	10.9	37.4	16.3
CS−, B3, F	−8.2	10.4	40.1	11.4
CS+, B3, S	6.7	12.8	53.5	15.6
CS−, B3, S	5.1	11.2	56.0	16.1
CS+, B3, F: low performance	–	–	29.4	10.3
CS−, B3, F: low performance	–	–	36.5	10.3
CS+, B3, S: low performance	–	–	45.1	14.7
CS−, B3, S: low performance	–	–	48.4	15.7

*Factors are: Stimulus (CS+, CS−), Block (Block 1: B1, Block 2: B2, Block 3: B3), Velocity (Fast: F, Slow: S)*.

**Figure 2:**
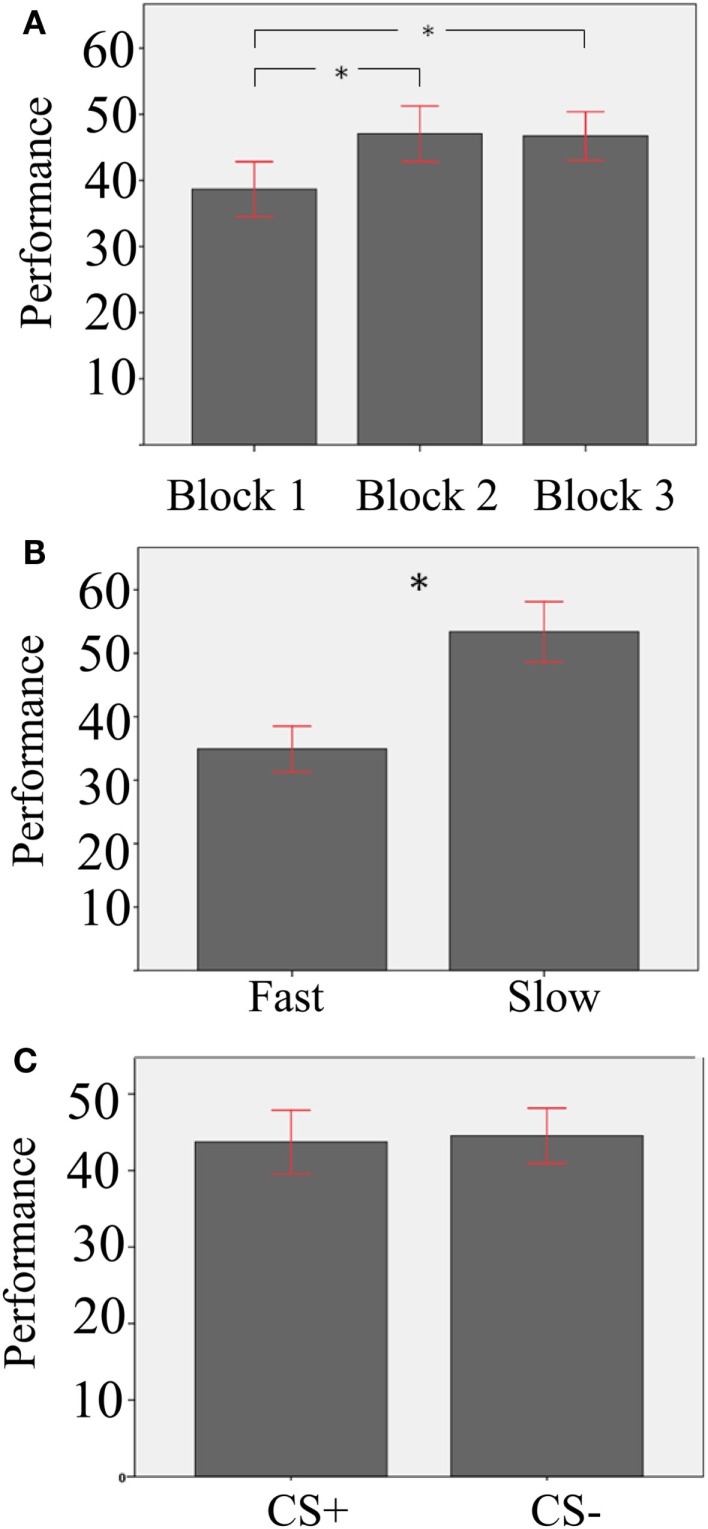
**Mean effects of the aggregated repeated-measures ANOVA with performance as dependent measure**. **(A)** Effect of Block; asterisks indicate significant differences at a Bonferroni-corrected threshold of 0.05; **(B)** Effect of Velocity; the asterisk indicates a significant difference **(C)** non-significant effect of Stimulus. Error bars show the 95% confidence interval.

In a second aggregated repeated-measure ANOVA analysis, we used the same factors as before, and performance, namely the percentage of winnings aggregated for each subject, as dependent variable (see Table 1 for means and SD in the different conditions). Main effects are shown in Figure 2. Results showed a main effect of Block [*F*(2,37) = 28.26, *p* = 0.000, $\eta_{p}^{2}=0.43$]. Paired-sample *T*-tests comparing Block 2 and Block 3 against Block 1 were significant [Block 1 vs. Block 2: *T*(37) = −6, *p* = 0.000; Block 1 vs. Block 3: *T*(37) = −6,3, *p* = 0.000; significance threshold Bonferroni-corrected], in line with the idea that the task was more routinized in the second and third blocks compared to the first one. In addition, participants performed better with Slow than Fast Velocity [*F*(1,37) = 91.65, *p* = 0.000, $\eta_{p}^{2}=0.71$]. A main effect of Stimulus was not present [*F*(1,37) = 0.59, *p* = 0.448]. However, we found a significant Stimulus-Velocity interaction [*F*(1,37) = 9.64, *p* = 0.004, $\eta_{p}^{2}=0.2$]. All other interactions were not significant [Stimulus-Block, *F*(2,74) = 1.1, *p* = 0.334; Velocity-Block, *F*(2,74) = 1.94, *p* = 0.152; Velocity-Block-Stimulus, *F*(2,74) = 2.04, *p* = 0.137].

**Figure 3:**
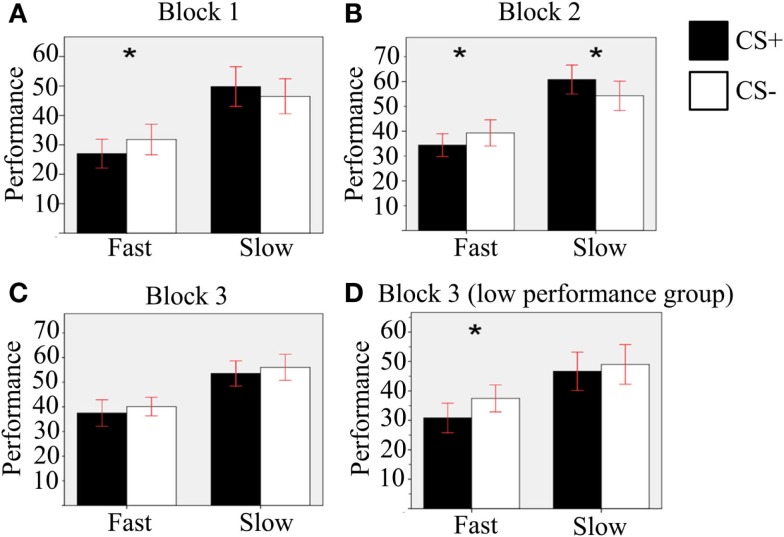
**Results of the orthogonal planned comparisons**. Comparison between CS+ and CS− condition with Fast and Slow velocities in **(A)** the first block; **(B)** the second block; **(C)** the third block; **(D)** for the Low-performance subgroup, in the third block. Error bars show the 95% confidence interval. Asterisks indicate significant differences.

To investigate our hypotheses specifically, we conducted orthogonal planned comparisons using paired-samples *T*-tests comparing CS+ and CS− trials across other conditions. We had an *a priori* hypothesis that performance in CS+ condition was worse than CS− condition with Fast Velocity, and vice versa with Slow Velocity, along all blocks (using one-tailed *T*-tests). This hypothesis derived from the expected role of TTD, which corresponded to Velocity in our paradigm. Results of orthogonal planned comparisons (Figure 3; see Table 1 for means and SD) confirmed that, in Blocks 1 and 2, performance was worse in CS+ compared to CS− condition with Fast Velocity [Block 1: *T*(37) = −1.8, *p* = 0.04, *r* = 0.28; Block 2: *T*(37) = −2.5, *p* = 0.008, *r* = 0.38]. This effect was not found in Block 3 [*T*(37) = −1.08, *p* = 0.144]. We found that performance was better in CS+ compared to CS− condition with Slow Velocity only in Block 2 [*T*(37) = −1.96, *p* = 0.029, *r* = 0.31], while we did not find such effect in the other blocks [Block 1: *T*(37) = 1.18, *p* = 0.125; Block 3: *T*(37) = −1.01, *p* = 0.159].

To further investigate the Pavlovian effect in the third block, we split the experimental sample in two subgroups using the median performance as a discriminative point (each group included 19 participants). Comparing CS+ to CS− condition in the third block for the Low-performance subgroup (see Figure 3D), performance was worse in the former than in the latter condition, with Fast Velocity [*T*(18) = −2.2, *p* = 0.022, *r* = 0.46]. No effect was found with Slow Velocity [*T*(18) = 0.11, *p* = 0.26].

### Control measures

We collected some control measures to ascertain that the effects we found on performance were genuine. Perceived shock intensity in the first block was not significantly different from the one in the second block [paired-samples *T*-test: *T*(37) = 0.77, *p* = 0.442]. Similarly, the perceived shock unpleasantness did not significantly vary across the two blocks [*T*(37) = 0.49, *p* = 0.631]. Overall, participants trusted the instructions that they would not have received any shock in the third block. Indeed, the mean VAS score (in a 0–10 range) about the strength of the belief that they would have not received any shock in the third block was 9.5, and no scores were under 8.

A possible explanation of the observed impairing effect of CS+ on performance with Fast Velocity, compared to either a null effect (Block 1) or an increasing effect (Block 2) with Slow Velocity, might be that participants, in the CS+ condition, adopted different strategies with different velocities. In other words, they could have chosen to press as precisely as possible with Slow Velocity, since avoiding shock was relatively easier in this condition. On the contrary, since avoiding shock was relatively harder with Fast Velocity, they could have chosen not to employ much effort in responding, but, rather, to concentrate in releasing the button as soon as possible. Contrary to our hypothesis of the interaction between Pavlovian and instrumental controllers, this possibility is in line with the idea that behavior was only instrumental in the experiment. If this was true, we would have observed that participants, with Fast Velocity, released the button before they did with Slow Velocity. To rule out this possibility, we compared the time spent pressing the button in CS+ trials with Fast Velocity and with Slow Velocity, for Block 1 and Block 2. Results of this analysis demonstrated that participants did not release the button differentially with the two velocities in CS+ trials, obtaining the same amount of shock [paired-sample *T*-tests, one-tailed; Block 1: *T*(37) = 0.43, *p* = 0.334; Block 2: *T*(37) = 0.54, *p* = 0.295]. This result rules out the possibility that behavior, with Fast Velocity, was strategically aimed at minimizing shock by releasing the button quickly, at the expense of performance.

### Between-subjects analysis

**Figure 4:**
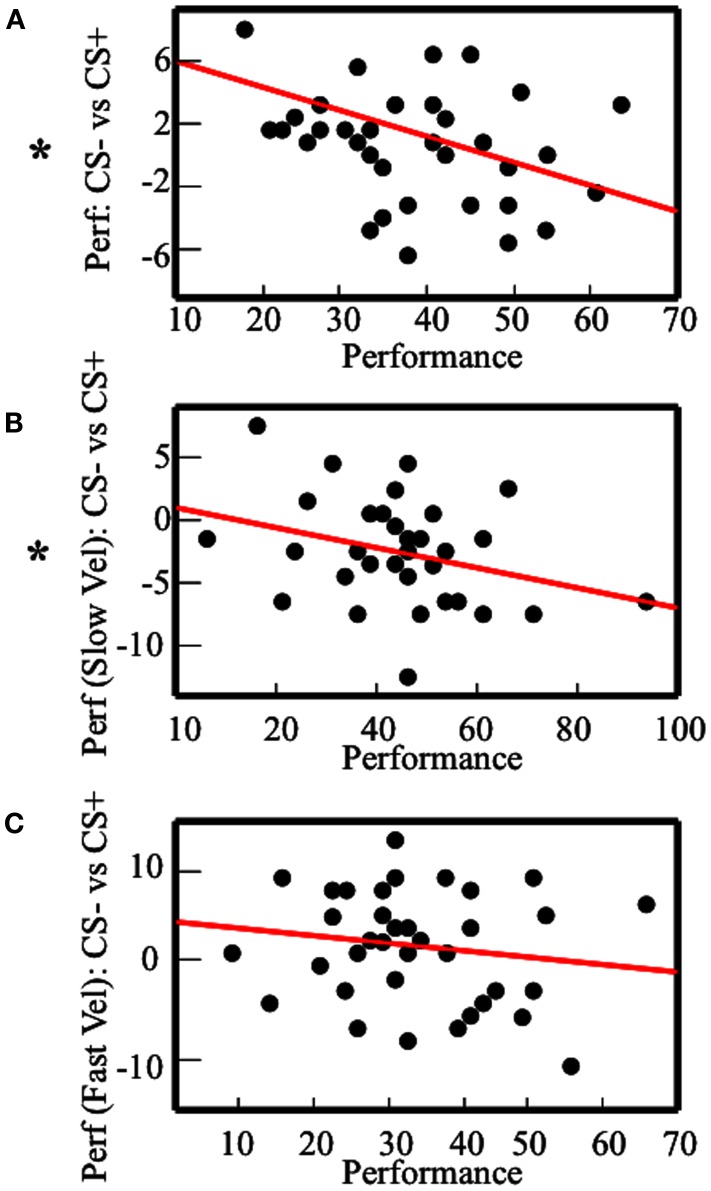
**Results of the between-subjects analysis**. Correlation between the performance and the performance with CS− minus the performance with CS+. The plots describe the analysis with: **(A)** the average performance; **(B)** the performance with Slow Velocity; **(C)** the performance with Fast Velocity. Asterisks indicate significant Pearson-*r*.

In the aggregated within-subjects analysis, we found a Stimulus-Velocity interaction effect on performance. As an inherent part of the paradigm, Velocity was associated with TTD. However, results showed that Velocity was also associated with CON (i.e., performance was better with Slow than Fast Velocity) and MV (i.e., participants collected more shocks with Fast Velocity than Slow Velocity). Therefore, from the previous analysis, it was not possible to assess the specific roles of TTD, CON, and MV as modulator variables of the effect of Pavlovian responses on instrumental performance. As a first way to test for an independent influence of CON, we devised a between-subjects analysis where we correlated the average performance with the difference in performance in CS− and CS+ conditions. The rationale of this analysis is that average performance can be considered as an index of each subject’s CON on the task, which is independent of Velocity, since all subjects had the same amount of Fast and Slow Velocity trials. Therefore, by comparing participants with bad performance (corresponding to low CON) and participants with better performance (corresponding to high CON) in respect with the effect of Pavlovian responses on performance, it was possible to test for a specific role of CON, independently of the role of Velocity. This analysis (Figure 4A) revealed an inverse correlation between general performance and difference between performance with CS− and CS+ (*r* = −0.36, *p* = 0.013, one-tailed). However, given that average performance was calculated by adding CS+ and CS− performances, this measure was not independent of CS− minus CS+ performance. This can create concerns about the interpretation of the obtained correlation, since the two correlated variables were dependent ones. In general, the correlation between *x* + *y* and *x* − *y*, where *x* and *y* are stochastic independent variables, should be zero when *x* and *y* have homogeneous variances. Therefore, to ensure that CS+ and CS− performances had homogeneous variances, we conducted a Levene’s Test, which resulted not significant [*F*(1,74) = 1.61, *p* = 0.2]. This supported the idea that the correlation between average performance and performance with CS− minus CS+ was effective, and was not an artifact effect. In addition, the correlation between performance and performance with CS− minus CS+ was analyzed for each of the two velocities (Figures 4B,C, respectively). With Slow Velocity, this correlation was significant (*r* = −0.32, *p* = 0.025, one-tailed). With Fast Velocity, it was not significant (*r* = −0.17, *p* = 0.14, one-tailed). Levene’s test was conducted also for the correlation with Slow Velocity, and it resulted not significant [*F*(1,74) = 0.46, *p* = 0.5].

### Trial-by-trial analysis

As a second method to disentangle the roles of TTD, CON, and MV, we devised a model-based trial-by-trial statistical analysis (Daw, 2011; see Appendix for details). Indeed, although TTD, MV, and CON overlapped in the aggregated data, they could be disentangled if we consider data on a trial-by-trial basis. To do this, we operationalized TTD, MV, and CON in different ways (see Appendix for details). Specifically, CON varied according to the recent winning history; MV varied according to the recent shock history; and TTD was assumed to correspond to the Stimulus × Velocity interaction. By comparing the independent effects of TTD, MV, and CON on trial-by-trial performance (a dichotomous variable whose values were “correct response” and “incorrect response”), it was possible to disentangle the specific role of each variable in the modulation of the influence of Pavlovian responses on performance.

We used the ANOVA model as the baseline model, and we built a pool of alternative models including different combinations of the ANOVA elements plus MV and CON (see Table 2). Overall, the trial-by-trial model-based analysis revealed that the model implementing the ANOVA model plus MV performed better than other models in predicting performance on a trial-by-trial basis. In addition, it is important to note that adding MV to the ANOVA model did not affect the significance of the STIM × VEL parameters. Thus, the effect of TTD, operationalized as the STIM × VEL interaction, appeared not to be influenced by the trial-by-trial changes in MV and CON. In sum, TTD and MV, contrary to CON, appeared to be independent modulator variables of the Pavlovian effect on performance on a trial-by-trial basis.

**Table 2:** **Labels and equations of the models**.

Model	LR	Formula	AIC	−LL
1	–	α	6826	6826
2	–	α + β · BLOCK	6824	6748
3	–	α + β · BLOCK + γ · VEL	6612	6460
4	–	α + β · BLOCK + γ · VEL + δ · STIM	6648	6420
5	–	α + β · BLOCK + γ · VEL + δ · STIM + ε · VEL · STIM	6671	6367
6	0.1	α + β · BLOCK + γ · VEL + ζ · MV	6636	6408
7	0.1	α + β · BLOCK + γ · VEL + δ · STIM + ζ · MV	6624	6320
8	0.1	α + β · BLOCK + γ · VEL + ε · VEL · STIM + ζ · MV	6616	6312
9	0.1	α + β · BLOCK + γ · VEL + δ · STIM + ε · VEL · STIM + ζ · MV	6576	6196
10	0.4	α + β · BLOCK + γ · VEL + ζ · MV	6631	6403
11	0.4	α + β · BLOCK + γ · VEL + δ · STIM + ζ · MV	6660	6356
12	0.4	α + β · BLOCK + γ · VEL + ε · VEL · STIM + ζ · MV	6621	6317
13	0.4	α + β · BLOCK + γ · VEL + δ · STIM + ε · VEL · STIM + ζ · MV	6649	6269
14	0.7	α + β · BLOCK + γ · VEL + ζ · MV	6633	6405
15	0.7	α + β · BLOCK + γ · VEL + δ · STIM + ζ · MV	6671	6367
16	0.7	α + β · BLOCK + γ · VEL + ε · VEL · STIM + ζ · MV	6640	6336
17	0.7	α + β · BLOCK + γ · VEL + δ · STIM + ε · VEL · STIM + ζ · MV	6679	6299
18	1	α + β · BLOCK + γ · VEL + ζ · MV	6640	6412
19	1	α + β · BLOCK + γ · VEL + δ · STIM + ζ · MV	6679	6375
20	1	α + β · BLOCK + γ · VEL + ε · VEL · STIM + ζ · MV	6657	6353
21	1	α + β · BLOCK + γ · VEL + δ · STIM + ε · VEL · STIM + ζ · MV	6694	6314
22	0.1	α + β · BLOCK + γ · VEL + η · CON · STIM	6648	6420
23	0.1	α + β · BLOCK + γ · VEL + δ · STIM + η · CON · STIM	6683	6379
24	0.1	α + β · BLOCK + γ · VEL + ε · VEL · STIM + η · CON · STIM	6685	6381
25	0.1	α + β · BLOCK + γ · VEL + δ · STIM + ε · VEL · STIM + η · CON · STIM	6680	6300
26	0.4	α + β · BLOCK + γ · VEL + η · CON · STIM	6647	6419
27	0.4	α + β · BLOCK + γ · VEL + δ · STIM + η · CON · STIM	6674	6370
28	0.4	α + β · BLOCK + γ · VEL + ε · VEL · STIM + η · CON · STIM	6679	6375
29	0.4	α + β · BLOCK + γ · VEL + δ · STIM + ε · VEL · STIM + η · CON · STIM	6686	6306
30	0.7	α + β · BLOCK + γ · VEL + η · CON · STIM	6643	6415
31	0.7	α + β · BLOCK + γ · VEL + δ · STIM + η · CON · STIM	6675	6371
32	0.7	α + β · BLOCK + γ · VEL + ε · VEL · STIM + η · CON · STIM	6672	6368
33	0.7	α + β · BLOCK + γ · VEL + δ · STIM + ε · VEL · STIM + η · CON · STIM	6692	6312
34	1	α + β · BLOCK + γ · VEL + η · CON · STIM	6642	6414
35	1	α + β · BLOCK + γ · VEL + δ · STIM + η · CON · STIM	6679	6375
36	1	α + β · BLOCK + γ · VEL + ε · VEL · STIM + η ·CON · STIM	6669	6365
37	1	α + β · BLOCK + γ · VEL + δ · STIM + ε · VEL · STIM + η · CON · STIM	6699	6319
38	0.1	α + β · BLOCK + γ · VEL + δ · STIM + ε · VEL · STIM + ζ · MV + λ · Shock*_n−1_*	6590	6134
39	0.1	α + β · BLOCK + γ · VEL + δ · STIM + ε · VEL · STIM + ζ · MV + μ · Outcome*_n−1_*	6601	6145
40	0.1	α + β · BLOCK + γ · VEL + δ · STIM + ε · VEL · STIM + ζ · MV + μ · Outcome*_n−1_* + λ · Shock*_n−1_*	6657	6125

*LR, learning rate of the model; AIC, Akaike information criterion; −LL, negative log-likelihood*.

## Discussion

The present study aimed to elucidate the Pavlovian-instrumental interaction in humans. Recent studies have investigated this issue, demonstrating that Pavlovian values influence instrumental behavior (Bray et al., 2008; Talmi et al., 2008; Huys et al., 2011). In particular, it has been shown that Pavlovian processes influence action and goal values. However, evidence in relation to a Pavlovian influence on instrumental performance is scarce. In other words, it is unclear whether and in which conditions Pavlovian responses facilitate, or interfere with, instrumental goals (Guitart-Masip et al., 2011). Following animal studies like the negative auto-maintenance paradigm (Williams and Williams, 1969), we aimed to investigate the Pavlovian influence on instrumental performance in humans, and the potential modulator variables of this influence.

To this aim, we studied human behavior in a simple sensorimotor task, comparing a condition in which a cue signaled that mistakes were punished with shock (CS+ condition) with a control condition in which a different cue signaled that mistakes were not punished (CS− condition). We studied different putative modulator variables of the Pavlovian impact on performance: amount of training, explicit shock expectancy, ball velocity (TTD), shock history (MV), and task difficulty (CON).

A first result of the within-subjects aggregated analysis indicated that Pavlovian stimuli did not have any effect on the distance (from the target-circle) at which participants pressed the button. However, the same analysis having performance as dependent variable revealed that, in Blocks 1 and 2, average performance was worse in CS+ than CS−with Fast Velocity. In the first part of the task (Block 1), performance with Slow Velocity was not different in CS+ and CS−, but, in the second part (Block 2), it was better in the former than in the latter. It is important to note that explicit shock expectancy was present in both Block 1 and Block 2. On the contrary, in the last part of the task (Block 3), shock was no more expected. In this condition, low average-performance participants (but not high-average performance ones) were worse in CS+ than CS−, with Fast Velocity.

A limit of the within-subjects aggregated analysis was that ball Velocity was associated with TTD, CON, and MV. Indeed, TTD was inherently associated with Velocity. In addition, participants performed better with Slow than Fast Velocity (CON), and received more shocks in Fast than Slow Velocity (MV). Therefore, the aggregated analysis did not allow us to detect the modulator independent contributes. To test for the independent roles of TTD, MV, and CON, we devised two methods: a between-subjects analysis and a trial-by-trial analysis. The between-subjects analysis showed that participants’ average performance, depending on participants’ CON, was correlated with the Pavlovian impairing effect. In other words, participants with bad performance, compared to participants with better performance, tended to perform worse in CS+ than CS− trials.

As a second method to disentangle the independent effects of the modulator variables, we conducted a trial-by-trial analysis. Indeed, TTD, MV, and CON could be operationalized in such a way that they varied independently on a trial-by-trial basis. This analysis showed that TTD and MV, contrary to CON, had independent effects on the Pavlovian influence on performance on a trial-by-trial basis. In particular, in relation to TTD, performance decreased with CS+ when the threat was nearest in time. In relation to MV, performance improved with CS+ after a shock had been collected in the previous CS+ trial. Overall, the between-subjects and the trial-by-trial analyses showed that TTD, CON, and MV had independent roles as modulator variables. However, these variables acted at different levels. Specifically, TTD and MV acted at a local level, since their influence was observed on a trial-by-trial basis. On the contrary, CON acted at a more global level. Indeed, its influence was observed at the between-subjects level only, suggesting that participants had a quite stable CON-related belief (independently of the recent success history), that modulated the Pavlovian impact on performance.

We interpret these results within a theoretical framework which views behavior as the output of the interaction between instrumental and Pavlovian controllers (Daw et al., 2005; Dayan and Seymour, 2008). According to this perspective, instrumental controllers select actions proportionally to their values, although each controller follows specific rules (Daw et al., 2005). In the present study, if only this process was active, performance should have been better in CS+ than CS− condition. However, as animal studies have shown, in many cases behavior is not only instrumental. Rather, reactive hard-wired responses to rewards and punishments either impair or strengthen the instrumental action efficacy (Dayan and Seymour, 2008).

In line with this view, we interpret the performance impairment in CS+ respect to CS− condition with Fast Velocity as the Pavlovian maladaptive influence on instrumental performance. Indeed, with Fast Velocity, people performed worse when expecting shock than no-shock. Consequently, they avoided less shocks than they could (as confirmed also by the analysis of the time spent pressing), plausibly against their intentions. This finding suggests that, similarly to the negative auto-maintenance paradigm in animals (Williams and Williams, 1969), Pavlovian responses can have maladaptive effects in humans too.

Alternative explanations of these findings are related to the “choking under pressure effect,” according to which people sometimes perform worse when facing high rewards and punishments. The “choking under pressure effect” has been given three different interpretations. The first one hypothesizes an inverted-U relationship between arousal and performance (Yerkes and Dodson, 1908; Neiss, 1988). The second one maintains that, in stressful situations, people choose to rely on controlled strategies during a task execution, with the idea that this choice is more advantageous compared to relying on more automatic mechanisms. However, this strategy actually would carry to performance decay, in line with studies showing that controlled strategies are less effective for highly practiced and automated tasks (Langer and Imber, 1979). Finally, a third interpretation of the “choking under pressure effect” maintains that stressful situations determine a narrowing of attention, which, in turn, would be detrimental for tasks requiring creativity and flexibility (Eisterbrook, 1959).

In general, the multicontroller framework is not incompatible with the standard interpretations of the “choking under pressure effect.” However, we believe that, for understanding our results, the multicontroller framework should be preferred to these interpretations. The general reason is that, in the present paradigm, the state of “pressure” was specifically manipulated through Pavlovian stimuli presentation, rather than through other mechanisms, as in standard “choking under pressure” experiments. Therefore, the state of “pressure” was reasonably mediated by Pavlovian mechanisms, and behavior was reasonably influenced by Pavlovian responses. In respect to the first model of the “choking under pressure effect,” it has been argued that the construct of arousal is quite ambiguous (Neiss, 1988). For example, it is not clear which arousal components would lead to performance increasing and decreasing. In addition, it is not clear how arousal is influenced by the environment, and which computational principles it follows. Therefore, we argue that referring to the multicontroller framework, whose features have been largely studied psychologically, neurally, and computationally, is more useful to interpret the present results. In respect to the second proposal related to the “choking under pressure effect” (Langer and Imber, 1979), this could have hypothesized that shock expectancy made participants rely on more controlled strategies, with maladaptive consequences. If this was true, we would have observed general performance decay with shock. On the contrary, decay was observed only with Fast Velocity, whereas performance increased with Slow Velocity (in the second block). This finding can hardly be reconciled with the idea that controlled strategies, triggered by shock, impaired performance. Finally, in relation to the third hypothesis on the “choking under pressure effect” (Eisterbrook, 1959), we argue that the present experimental task was very simple and repetitive, and distracters were absent. Therefore, the narrowing of attention, advocated by this hypothesis as responsible for performance decay under pressure, can hardly explain our observations.

The between-subjects and the trial-by-trial analyses showed that TTD, CON, and MV had independent roles in modulating the effects of Pavlovian responses on performance. Past research has indicated that temporal and spatial distances modulate aversive behavior (Fanselow and Lester, 1988; Blanchard and Blanchard, 1989). Therefore, in relation to the present study, TTD, which co-varied with ball velocity, reasonably modulated the Pavlovian influence on performance. However, ball velocity could have exerted its effect via other mechanisms. A possibility is that Velocity *per se*, rather than TTD, influenced the Pavlovian effect on performance. This possibility cannot be ruled out by the present experiment, and deserves further investigation.

The between-subjects analysis suggests that average performance, linked to participant’s CON, inversely correlated with the impairing Pavlovian effect on performance. This is in line with evidence indicating that CON modulates Pavlovian activation. In particular, it has been shown that low CON increases fear conditioned responses (Mineka et al., 1984). The finding that the trial-by-trial analysis did not reveal any local effect of CON is apparently at odds with this interpretation. However, learned-helplessness studies suggest that CON is quite stable, and is not influenced by local performance changes (Mineka and Hendersen, 1985; Maier and Watkins, 2005). This could explain why CON effects could not be detected on a trial-by-trial basis, but emerged when comparing participants with different average performance.

Finally, the trial-by-trial analysis indicated an independent effect of MV. This variable depended on past shocks. We first hypothesized that MV impaired performance, by enhancing the Pavlovian maladaptive activation. Alternatively, MV could have improved performance, by increasing the value of the goal of avoiding the punishment. The trial-by-trial analysis, showing that performance increased in CS+ after a shock had been collected in the previous CS+ trial, supported the latter hypothesis.

We also tested whether Pavlovian effects on performance were detected even without explicit shock expectancy. With this regard, Pavlovian impairing effects with Fast Velocity emerged also in extinction, namely in a last block without shocks (in which participants knew about the new contingency). Noteworthy, this effect was found only in low average-performance participants. These results are in accordance with previous findings showing that the skin conductance conditioned response can be detected even without explicit shock expectancy (Schell et al., 1991; Lipp and Edwards, 2002). In relation to these and our findings, it is possible that two distinct processes, one model-based and the other model-free, as defined in reinforcement-learning literature (Sutton and Barto, 1998; Daw et al., 2005; Rigoli et al., 2011; Solway and Botvinick, 2012), are involved in Pavlovian learning. When the explicit belief about the stimulus-shock contingency is reversed by verbal instructions, as in the third block, the model-based Pavlovian process would be quickly updated. On the contrary, the model-free process would not be affected by verbal instructions, and would continue to trigger directly a CR every time a CS+ appears. The fact that the maladaptive Pavlovian influence was found only in low average-performance participants could be due to the modulatory effect of CON (low average performance corresponded to low CON) on the model-free Pavlovian activation.

Another issue regards the differential Pavlovian impact on goal-directed and habitual mechanisms. The fact that performance improved in Block 2 and 3, compared to Block 1, allowed us to assume that, in Blocks 2 and 3, the habitual system was more active than in Block 1. The Pavlovian impairing effect with Fast Velocity emerged both in Blocks 1 and 2, associated to goal-directed and habitual mechanisms, respectively. On the contrary, the enhancing effect of CS+ on performance with Slow Velocity emerged in Block 2 only. A possible explanation of this evidence is that over-learned reactions, linked to habitual control, are immune to impairing Pavlovian effects in slow trials. Alternatively, CS+ could increase performance in over-learned tasks, contrary to non-over-learned ones, in slow trials. However, CON might be actually responsible for the differential Pavlovian effects on goal-directed and habitual mechanisms. Indeed, CON was lower in Block 1 than Block 2, since average performance increased along blocks.

In relation to the present results, an important aspect regards the specific nature of the CR, and the level at which it influenced instrumental performance. Following previous literature, three different hypotheses can be formulated, based on the idea of a conflict between an instrumental motor command and a co-occurring CR. The hypotheses differ with respect to the nature of the CR and the level of its influence. A first hypothesis is that, in line with neurobiological evidence (Butler et al., 2007), the CR corresponded to a specific motor response competing against a co-occurrent instrumental motor command. According to this interpretation, in the context of our experiment, the instrumental motor response of pressing the button at the right time could be impaired by a co-active specific CR of withdrawing the finger, associated with the painful shock delivered to the finger itself. The second hypothesis is that an aversive CS+ triggered a general motor inhibition reaction, leading to instrumental impairment (Gray, 1982; Crockett et al., 2009; Guitart-Masip et al., 2011). Finally, the third hypothesis is that a non-specific CR (e.g., trembling) impaired the precision of an instrumental motor command (Mobbs et al., 2009). The precision of a motor command can be defined as the noise of the actual behavior with respect to a planned motor command. In the context of our experiment, this noise could be inflated by a non-specific CR, leading to performance decrease. These three hypotheses make different predictions in respect to our experimental results. According to the first two hypotheses, it was expected that participants pressed at a larger distance to the target in CS+ than CS−. However, we did not find any Stimulus effect on distance, not even considering its interaction with Velocity or Block. This result is more consistent with the third hypothesis, postulating a non-specific CR, such as trembling, affecting instrumental motor precision.

A final aspect regards the associative relationships underlying conditioning in our experiment. Each trial included the following stimuli: the circle, the moving ball, and the visual feedback (plus the shock in some cases). The visual feedback and the shock worked as US and, in our analysis, we assumed that the circle worked as CS+. However, an alternative possibility is that the moving ball worked actually as CS+, whereas the circle worked as an occasion setter. Associative learning theories distinguish between CSs and occasion setters, which would follow different associative processes. In particular, CSs have a direct relationship with USs, whereas occasion setters determine the condition in which CSs are associated with USs (Holland, 1992). A limit of the paradigm we used is that it did not allow to ascertain whether either the circle or the moving ball worked as CS+. However, we argue that this limit does not hinder the present findings, since they are related to the effects of Pavlovian responses on performance. In other words, the CR influence on performance, modulated by the variables investigated, is not affected by the fact that the circle or the moving ball worked as CS+. However, future research should understand which CSs can produce CRs influencing instrumental performance. Another important aspect of the present study regards its generalizability. Indeed, the Pavlovian effects (and their modulators) found here could be related to the task used, which required a simple and precise motor execution. It is possible that Pavlovian mechanisms have maladaptive effects only in some kinds of task, whereas they might have neutral, or even positive, effects in other kinds. Further investigation is needed for the study of Pavlovian influence in other tasks and conditions.

### The neurobiology of the interaction between Pavlovian and instrumental controllers

In this section, we describe the neurobiology of the Pavlovian-instrumental interaction and relate it to the present findings. Evidence suggests that different motivational systems (goal-directed, habitual, and Pavlovian) involve specific neural substrates (Balleine and Dickinson, 1998; Yin et al., 2008; Balleine and O’Doherty, 2009; Glascher et al., 2010; Guitart-Masip et al., 2011; Huys et al., 2011; Pezzulo and Rigoli, 2011; Simon and Daw, 2011; Wunderlich et al., 2012). The importance of studying the neural substrates of the interaction amongst controllers has been recently stressed (Balleine and O’Doherty, 2009; Bornstein and Daw, 2011), since these interactions are poorly understood, especially regarding the instrumental-Pavlovian one. In relation to this, research has mostly focused on the Pavlovian effects on action value and general motor reactivity.

With regard to action value, orbitofrontal cortex (Padoa-Schioppa and Assad, 2006), amygdala (Schoenbaum et al., 2003), and striatum (O’Doherty et al., 2004) have been shown to encode action values, although these structures are differentially recruited by the goal-directed and the habitual controllers (Pennartz et al., 2011; Wunderlich et al., 2012). At the same time, amygdala and ventral striatum have been shown to encode Pavlovian values (O’Doherty et al., 2004; Yin et al., 2008; Balleine and O’Doherty, 2009). In line with this evidence, recent findings suggest that amygdala and basal ganglia, where Pavlovian and instrumental value computations overlap, are crucial for Pavlovian-instrumental interactions (Bray et al., 2008; Talmi et al., 2008).

Other studies have focused on the Pavlovian effects on general motor reactivity (Gray, 1982; Crockett at al., 2009; Guitart-Masip et al., 2011). Dopamine and serotonin have been extensively reported as modulators of motivation and vigor (Niv et al., 2007; Boureau and Dayan, 2010). Recent studies have suggested that these neurotransmitters, particularly in the striatum, are responsible for excitatory and inhibitory Pavlovian effects on motor reactivity (Talmi et al., 2008; Crockett at al., 2009; Guitart-Masip et al., 2011).

In addition to action value and general motor reactivity, Pavlovian mechanisms might influence also other aspects of instrumental behavior, which should be considered by future research. For instance, Pavlovian and instrumental controllers might interact at specific motor levels, activating parallel motor neural processes. With this regard, the periaqueductal gray matter triggers automatic Pavlovian motor responses (Keay and Bandler, 2001), whereas cortical motor areas (the motor, pre-motor, and supplemental motor areas), together with basal ganglia, produce instrumental motor outputs (Balleine and Dickinson, 1998; Yin et al., 2008; Balleine and O’Doherty, 2009). A Pavlovian stimulus might activate specific motor outputs both in the periaqueductal gray matter and in instrumental motor areas.

A second underexplored possibility in relation to the Pavlovian-instrumental interaction is that Pavlovian mechanisms impact on the precision of a motor execution. The motor precision is partially independent of rapidity and motor reactivity. The present findings, showing an effect on precision and no effect on rapidity, suggest that Pavlovian stimuli can have a specific impact on the motor execution precision. At the neural level, neurotransmitters as dopamine, serotonin, and noradrenaline are known to modulate the executive processes, and they might impact on the motor execution precision (Niv et al., 2007; Boureau and Dayan, 2010). In addition, structures such as amygdala and striatum have modulatory effects on executive processes, and might also be involved in this context (Davis, 1992; Fanselow, 1994).

A final consideration regards the neural underpinnings of MV, TTD, and CON, indicated by the present study as modulating the instrumental-Pavlovian interaction. After a shock, the goal of avoiding punishment in the future (MV in our analysis) increased, leading to performance improvement. Goal values are encoded by orbitofrontal cortex and amygdala (Balleine and O’Doherty, 2009). In relation to threat distance, it is well known that emotional stimuli are partially processed by distinct neural structures compared to neutral stimuli (Vuilleumier and Driver, 2007). On the base of this, an intriguing possibility is that perceptual information on temporal and spatial threat distances preferentially activates amygdala, which is crucial for elaborating emotional stimuli. Moreover, perceptual information on threat distance might reach the amygdaloid nuclei through the direct thalamo-amygdala pathway, which has been hypothesized to be recruited by highly salient emotional stimuli (Vuilleumier and Driver, 2007). Finally, in relation to CON, evidence indicates that serotoninergic dorsal raphé nuclei and ventromedial prefrontal cortex implement CON at the neural level (Amat et al., 2005; Maier and Watkins, 2005). In particular, dorsal raphé nuclei would lead to uncontrollability effects, whereas ventromedial prefrontal cortex, by inhibiting the former structure, would oppose to those effects.

## Conclusion

The study of the interaction between different motivational controllers is fundamental for understanding decision-making. On this basis, we investigated the Pavlovian-instrumental interaction, particularly underexplored in humans. Similarly to animal studies, the present findings support the view that Pavlovian responses impact on instrumental performance in humans too, and can produce misbehavior. In addition, amount of experience, shock expectancy, threat distance, punishment history, and task difficulty modulated the effect of Pavlovian responses on performance.

The Pavlovian-instrumental interaction could underlie some forms of irrationality in decision-making. Indeed, although Pavlovian mechanisms are possibly adaptive in most situations, nonetheless, in irrational decision-making, they might influence behavior in a way that is not congruent with the subject’s goals. However, it does not necessarily follow that, in these cases, Pavlovian mechanisms are irrational or non-optimal in absolute terms. Rather, Pavlovian and instrumental controllers might just follow different optimality criteria. In particular, instrumental controllers would follow optimality in ontogenetic terms, in the sense that they might be guided by reward maximization on the base of the organism’s experience. On the contrary, Pavlovian controllers would follow optimality in phylogenetic terms, in the sense that they might be guided by reward maximization on the base of the specie’s experience (Dayan et al., 2006). Research on this topic could help elucidating important phenomena, apparently irrational, in economics, social behavior, and psychopathology (Dayan and Seymour, 2008).

## Conflict of Interest Statement

The authors declare that the research was conducted in the absence of any commercial or financial relationships that could be construed as a potential conflict of interest.

## Appendix

### The trial-by-trial model-based analysis

We compared different trial-by-trial models of data, including different combinations of the ANOVA factors plus CON and MV. In order to operationalize MV and CON, we adopted a temporal-difference algorithm. This algorithm is a standard way for modeling variables depending on the history of motivational experience, like MV and CON. Indeed, MV (corresponding to the learned motivational values) depends on the history of past shocks, whereas CON (corresponding to the learned belief of performing well) depends on the history of success.

We considered the two Stimuli (CS+ and CS−) as distinct conditions for the computation of MV. Similarly, we considered the two Velocities (Fast and Slow) as distinct conditions for the computation of CON. Specifically, the MV associated to each trial depended on the MV associated to the previous trial of the same Stimulus condition, updated according to the shock-related outcome obtained in that previous trial. The following temporal-difference algorithm was used as learning rule (Sutton and Barto, 1998):

$$\text{M}{\text{V}}_{\text{STIM}n+1}=\text{M}{\text{V}}_{\text{STIM}n}+\text{LR}\cdot \left(R-\text{M}{\text{V}}_{\text{STIM}n}\right)$$

Where *R* represented the shock outcome of the trial (*R* = 0 if shock was avoided; *R* = −1 if shock was collected), LR represented the learning rate (LR), and the starting MV value was set to zero. It is important to note that, according to this learning mechanism, MV = 0 in CS− condition. In relation to CON, we operationalized it as a number comprised between 1 (when the participant has the maximum control on the environment) and zero (when the participant has not control at all). The CON associated to each trial depended on the CON associated to the previous trial belonging to the same Velocity condition, updated according to the outcome obtained in that previous trial. The following temporal-difference algorithm was used as learning rule (Sutton and Barto, 1998):

$$\text{CO}{\text{N}}_{\text{VEL}n+1}=\text{CO}{\text{N}}_{\text{VEL}n}+\text{LR}\cdot \left(B-\text{CO}{\text{N}}_{\text{VEL}n}\right)$$

Where *B* represented the outcome of the trial (*B* = 1 if the trial was won; *B* = 0 if the trial was lost), LR represented the learning rate, and the starting CON value was set to 0.5. We used this starting value because, in absence of any clue on the participants’ prior CON, it assigns the same probability (0.5) to the two possible outcomes (winning and losing).

We delimited the analysis to Blocks 1 and 2. To represent the trial-by-trial dynamics, we used logistic regression, which is based on a linear equation used to compute the probability of an outcome of a dichotomous variable, in our case the probability of winning at a given trial:

$$p(B=1)=\frac{1}{1+e^{\left(-\text{MODEL}\right)}}$$

Model parameters were inferred using the Least Square Method. The goodness of each model was estimated through the Akaike information criterion (AIC) index, and the parameters were assumed to be random variables across subjects, and were tested using independent-samples *T*-tests. The different models are shown in Table 2. Model 5 corresponds to the ANOVA model used for the analysis of the aggregated data, and was used as baseline model:

MODEL5=α+β⋅BLOCK + γ⋅VEL + δ⋅STIM + ε⋅VEL⋅STIM

In a pool of further models, we included also either MV or CON (multiplied by STIM in this latter case), as operationalized above. In addition, we tested different LRs (0.1; 0.4; 0.7; 1) for updating MV and CON in the models implementing these variables.

On the basis of the AIC index, no models implementing CON were better than the ANOVA model. On the contrary, some models implementing MV had a lower AIC compared to the ANOVA model. Overall, the model implementing the ANOVA model plus MV with a LR = 0.1 (model 9) resulted the best one (AIC = 6576). This result was corroborated also by the log-likelihood ratio test comparing the ANOVA model and model 9, made upon the mean negative log-likelihood across subjects [χ(1) = 12; *p* = 0.000]. Finally, *T*-tests indicated that all model 9 parameters were statistically significant [two-tailed independent-samples *T*-tests: β : *T*(37) = 2.58, *p* = 0.01; γ : *T*(37) = −4.48, *p* = 0.000; *δ* : *T*(37) = 3.72, *p* = 0.000; ε : *T*(37) = −4.76, *p* = 0.000; ζ : *T*(37) = −6.14, *p* = 0.000]. Importantly, the MV parameter was negative, indicating that performance increased with the increasing of the number of past shocks.

As a final step of the trial-by-trial analysis, we assessed the effect of a shock or an error collected in the previous trial, by including in the model the shock-related outcome and/or the outcome collected in the previous trial (model 38, 39, and 40). AIC indexes indicated that all these models performed worse than model 9.

## References

[B1] Amat J. Baratta M. Paul E. Bland S. Watkins L. Maier S. (2005). Medial prefrontal cortex determines how stressor controllability affects behavior and dorsal raphé nucleus. Nat. Neurosci. 8, 365–371 10.1038/nn1399 15696163 PMC13675003

[B2] Balleine B. Dickinson A. (1998). Goal-directed instrumental action: contingency and incentive learning and their cortical substrates. Neuropharmacology 37, 407–419 10.1016/S0028-3908(98)00033-1 9704982

[B3] Balleine B. O’Doherty J. (2009). Human and rodent homologies in action control: corticostriatal determinants of goal-directed and habitual action. Neuropsychopharmacology 35, 48–69 10.1038/npp.2009.131 19776734 PMC3055420

[B4] Blake D. Strata F. Churchland A. Merzenich M. (2002). Neural correlates of instrumental learning in primary auditory cortex. Proc. Natl. Acad. Sci. U.S.A. 99, 10114 10.1073/pnas.092278099 12119383 PMC126633

[B5] Blanchard R. Blanchard D. (1989). Attack and defense in rodents as ethoexperimental models for the study of emotion. Prog. Neuropsychopharmacol. Biol. Psychiatry 13:S3–S14 10.1016/0278-5846(89)90105-X 2694228

[B6] Bornstein A. M. Daw N. (2011). Multiplicity of control in the basal ganglia: computational roles of striatal subregions. Curr. Opin. Neurobiol. 21, 374–380 10.1016/j.conb.2011.02.009 21429734 PMC3269306

[B7] Boureau Y. Dayan P. (2010). Opponency revisited: competition and cooperation between dopamine and serotonin. Neuropsychopharmacology 36, 74–97 10.1038/npp.2010.151 20881948 PMC3055522

[B8] Bray S. Rangel A. Shimojo S. Balleine B. O’Doherty J. P. (2008). The neural mechanisms underlying the influence of Pavlovian cues on human decision making. J. Neurosci. 28, 5861 10.1523/JNEUROSCI.0897-08.2008 18509047 PMC6670800

[B9] Breland K. Breland M. (1966). Animal Behavior. Oxford: Macmillan

[B10] Brown P. Jenkins H. (1968). Auto-shaping of the pigeon’s key-peck. J. Exp. Anal. Behav. 11, 1 10.1901/jeab.1968.11-1 5636851 PMC1338436

[B11] Butler T. Pan H. Tuescher O. Engelien A. Goldstein M. Epstein J. (2007). Human fear-related motor neurocircuitry. Neuroscience 150, 1–7 10.1016/j.neuroscience.2007.09.048 17980493

[B12] Colwill R. Rescorla R. (1988). Associations between the discriminative stimulus and the reinforcer in instrumental learning. J. Exp. Psychol. Anim. Behav. Process. 14, 155 10.1037/0097-7403.14.2.155

[B13] Crockett M. J. Clark L. Robbins T. W. (2009). Reconciling the role of serotonin in behavioral inhibition and aversion: acute tryptophan depletion abolishes punishment-induced inhibition in humans. J. Neurosci. 29, 11993 10.1523/JNEUROSCI.2513-09.2009 19776285 PMC2775933

[B14] Davis M. (1992). The role of the amygdala in fear and anxiety. Annu. Rev. Neurosci. 15, 353–375 10.1146/annurev.ne.15.030192.002033 1575447

[B15] Daw N. (2011). “Trial-by-trial data analysis using computational models,” in Decision Making, Affect, and Learning: Attention and Performance XXIII, eds Delgado M. R. Phelps E. A. Robbins T. W. (Oxford: University Press), 3–38

[B16] Daw N. Niv Y. Dayan P. (2005). Uncertainty-based competition between prefrontal and dorsolateralstriatal systems for behavioral control. Nat. Neurosci. 8, 1704–1711 10.1038/nn1560 16286932

[B17] Dayan P. Niv Y. Seymour B. Daw N. D. (2006). The misbehavior of value and the discipline of the will. Neural Netw. 19, 1153–1160 10.1016/j.neunet.2006.03.002 16938432

[B18] Dayan P. Seymour B. (2008). “Values and actions in aversion,” in Neuroeconomics: Decision Making and the Brain, eds Glimcher P. W. Fehr E. Rangel A. Camerer C. Poldrack R. A. (New York: Academic Press), 175–191

[B19] Dickinson A. Pearce J. (1977). Inhibitory interactions between appetitive and aversive stimuli. Psychol. Bull. 84, 690 10.1037/0033-2909.84.4.690

[B20] Doyon J. Penhune V. Ungerleider L. G. (2003). Distinct contribution of the cortico-striatal and cortico-cerebellar systems to motor skill learning. Neuropsychologia 41, 252–262 10.1016/S0028-3932(02)00158-6 12457751

[B21] Eisterbrook J. A. (1959). The effect of emotion on cue utilization and the organization of behavior. Psychol. Rev. 66, 183–201 10.1037/h0047707 13658305

[B22] Estes W. Skinner B. (1941). Some quantitative properties of anxiety. J. Exp. Psychol. 29, 390 10.1037/h0062283

[B23] Fanselow M. (1994). Neural organization of the defensive behavior system responsible for fear. Psychon. Bull. Rev. 1, 429–438 10.3758/BF03210947 24203551

[B24] Fanselow M. Lester L. (1988). “A functional behavioristic approach to aversively motivated behavior: predatory imminence as a determinant of the topography of defensive behavior,” in Evolution and Learning, eds Bolles R. C. Beecher M. D. (Hillsdale, NJ: Lawrence Erlbaum Associates, Inc.), 185–211

[B25] Glascher J. Daw N. Dayan P. O’Doherty J. (2010). States versus rewards: dissociable neural prediction error signals underlying model-based and model-free reinforcement learning. Neuron 66, 585–595 10.1016/j.neuron.2010.04.016 20510862 PMC2895323

[B26] Gray J. (1982). The Psychology of Fear and Stress. Cambridge: University Press

[B27] Guitart-Masip M. Fuentemilla L. Bach D. R. Huys Q. J. M. Dayan P. Dolan R. J. (2011). Action dominates valence in anticipatory representations in the human striatum and dopaminergic midbrain. J. Neurosci. 31, 7867 10.1523/JNEUROSCI.6376-10.2011 21613500 PMC3109549

[B28] Hershberger W. (1986). An approach through the looking-glass. Learn. Behav. 14, 443–451 10.3758/BF03200092

[B29] Holland P. (2004). Relations between Pavlovian-instrumental transfer and reinforcer devaluation. J. Exp. Psychol. Anim. Behav. Process. 30, 104 10.1037/0097-7403.30.2.104 15078120

[B30] Holland P. C. (1992). “Occasion setting in Pavlovian conditioning,” in The Psychology of Learning and Motivation, ed. Medin D. L. (New York: Academic Press), 69–125

[B31] Hull C. (1943). Principles of Behavior. New York: Appleton-Century-Crofts

[B32] Huys Q. Cools R. Golzer M. Friedel E. Heinz A. Dolan R. Dayan P. (2011). Disentangling the roles of approach, activation and valence in instrumental and Pavlovian responding. PLoS Comput. Biol. 7, e1002028 10.1371/journal.pcbi.1002028 21556131 PMC3080848

[B33] Huys Q. Dayan P. (2009). A Bayesian formulation of behavioral control. Cognition 113, 314–328 10.1016/j.cognition.2009.01.008 19285311

[B34] Keay K. Bandler R. (2001). Parallel circuits mediating distinct emotional coping reactions to different types of stress. Neurosci. Biobehav. Rev. 25, 669–678 10.1016/S0149-7634(01)00049-5 11801292

[B35] Langer L. J. Imber L. G. (1979). When practice makes imperfect: the debilitating effects of overlearning. J. Pers. Soc. Psychol. 37, 2014–2024 10.1037/0022-3514.37.11.2014 521900

[B36] Lipp O. V. Edwards M. S. (2002). Effect of instructed extinction on verbal and autonomic indices of Pavlovian learning with fear-relevant and fear-irrelevant conditioned stimuli. J. Psychophysiol. 16, 176–186 10.1027//0269-8803.16.3.176

[B37] Loewenstein G. F. O’Donoghue T. (2004). Animal Spirits: Affective and Deliberative Processes in Economic Behavior. Available at SSRN: http://ssrn.com/abstract=539843 or http://dx.doi.org/10.2139/ssrn.539843

[B38] Mackintosh N. (1983). Conditioning and Associative Learning. New York: Oxford University Press

[B39] Maier S. Watkins L. (2005). Stressor controllability and learned helplessness: the roles of the dorsal raphé nucleus, serotonin, and corticotropin-releasing factor. Neurosci. Biobehav. Rev. 29, 829–841 10.1016/j.neubiorev.2005.03.021 15893820

[B40] McNaughton N. Corr P. (2004). A two-dimensional neuropsychology of defense: fear/anxiety and defensive distance. Neurosci. Biobehav. Rev. 28, 285–305 10.1016/j.neubiorev.2004.03.005 15225972

[B41] Mineka S. Cook M. Miller S. (1984). Fear conditioned with escapable and inescapable shock: Effects of a feedback stimulus. J. Exp. Psychol. Anim. Behav. Process. 10, 307 10.1037/0097-7403.10.3.307

[B42] Mineka S. Hendersen R. (1985). Controllability and predictability in acquired motivation. Annu. Rev. Psychol. 36, 495–529 10.1146/annurev.ps.36.020185.002431 3919637

[B43] Mobbs D. Marchant J. Hassabis D. Seymour B. Tan G. Gray M. Petrovic P. Dolan R. Frith C. (2009). From threat to fear: the neural organization of defensive fear systems in humans. J. Neurosci. 29, 12236 10.1523/JNEUROSCI.2378-09.2009 19793982 PMC2782300

[B44] Morse W. Mead R. Kelleher R. (1967). Modulation of elicited behavior by a fixed-interval schedule of electric shock presentation. Science 157, 215 10.1126/science.157.3785.215 17806273

[B45] Neiss R. (1988). Reconceptualizing Arousal: Psychological States in Motor Performance. Psychol. Bull. 103, 345–366 10.1037/0033-2909.103.3.345 3289072

[B46] Niv Y. Daw N. Joel D. Dayan P. (2007). Tonic dopamine: opportunity costs and the control of response vigor. Psychopharmacology (Berl.) 191, 507–520 10.1007/s00213-006-0502-4 17031711

[B47] O’Doherty J. Dayan P. Schultz J. Deichmann R. Friston K. Dolan R. (2004). Dissociable roles of ventral and dorsal striatum in instrumental conditioning. Science 304, 452 10.1126/science.1094285 15087550

[B48] Overmier J. B. Bull J. A. Pack K. (1971). On instrumental response interaction as explaining the influences of Pavlovian CS+s upon avoidance behavior. Learn. Motiv. 2, 103–112 10.1016/0023-9690(71)90001-4

[B49] Padoa-Schioppa C. Assad J. (2006). Neurons in the orbitofrontal cortex encode economic value. Nature 441, 223–226 10.1038/nature04676 16633341 PMC2630027

[B50] Pennartz C. M. Ito R. Verschure P. F. Battaglia F. P. Robbins T. W. (2011). The hippocampal-striatal axis in learning, prediction and goal-directed behavior. Trends Neurosci. 34, 548–559 10.1016/j.tins.2011.08.001 21889806

[B51] Pezzulo G. Castelfranchi C. (2009). Thinking as the control of imagination: a conceptual framework for goal-directed systems. Psychol. Res. 73, 559–577 10.1007/s00426-009-0241-3 19347359

[B52] Pezzulo G. Rigoli F. (2011). The value of foresight: How prospection affects decision-making. Front. Decis. Neurosci. 5:79 10.3389/fnins.2011.00079 PMC3129535 21747755

[B53] Pleger B. Blankenburg F. Ruff C. Driver J. Dolan R. (2008). Reward facilitates tactile judgments and modulates hemodynamic responses in human primary somatosensory cortex. J. Neurosci. 28, 8161 10.1523/JNEUROSCI.1093-08.2008 18701678 PMC2682779

[B54] Rescorla R. Solomon R. (1967). Two-process learning theory: relationships between Pavlovian conditioning and instrumental learning. Psychol. Rev. 74, 151 10.1037/h0024109 5342881

[B55] Rigoli F. Pavone E. Pezzulo G. (2011). “Interaction of goal-directed and Pavlovian systems in aversive domains,” in Proceedings of CogSci 2011, Boston

[B56] Schell A. M. Dawson M. E. Marinkovic K. (1991). Effects of potentially phobic conditioned stimuli on retention, reconditioning, and extinction of the conditioned skin conductance response. Psychophysiology 28, 140–153 10.1111/j.1469-8986.1991.tb00403.x 1946880

[B57] Schoenbaum G. Setlow B. Saddoris M. Gallagher M. (2003). Encoding predicted outcome and acquired value in orbitofrontal cortex during cue sampling depends upon input from basolateral amygdala. Neuron 39, 855–867 10.1016/S0896-6273(03)00474-4 12948451

[B58] Simon D. Daw N. (2011). Neural correlates of forward planning in a spatial decision task in humans. J. Neurosci. 31, 5526 10.1523/JNEUROSCI.3772-11.2011 21471389 PMC3108440

[B59] Solway A. Botvinick M. M. (2012). Goal-directed decision making as probabilistic inference: a computational framework and potential neural correlates. Psychol. Rev. 119, 120–154 10.1037/a0026435 22229491 PMC3767755

[B60] Sutton R. Barto A. (1998). Reinforcement Learning: An Introduction. Cambridge: University Press

[B61] Talmi D. Seymour B. Dayan P. Dolan R. J. (2008). Human Pavlovian-instrumental transfer. J. Neurosci. 28, 360 10.1523/JNEUROSCI.4028-07.2008 18184778 PMC2636904

[B62] Vuilleumier P. Driver J. (2007). Modulation of visual processing by attention and emotion: window son causal interactions between human brain regions. Philos. Trans. R. Soc. Lond. B Biol. Sci. 362, 837 10.1098/rstb.2007.2092 17395574 PMC2430001

[B63] Williams D. Williams H. (1969). Auto-maintenance in the pigeon: sustained pecking despite contingent non-reinforcement. J. Exp. Anal. Behav. 12, 511 10.1901/jeab.1969.12-511 16811370 PMC1338642

[B64] Wunderlich K. Dayan P. Dolan R. (2012). Mapping value-based planning and extensively trained choice in the human brain. Nat. Neurosci. 15, 786–793 10.1038/nn.3068 22406551 PMC3378641

[B65] Yerkes R. M. Dodson J. D. (1908). The Relationship of Strength of Stimulus to Rapidity of Habit-Formation. J. Comp. Neurol. Psychol. 18, 459–482 10.1002/cne.920180503

[B66] Yin H. Ostlund S. Balleine B. (2008). Reward-guided learning beyond dopamine in the nucleus accumbens: the integrative functions of cortico-basal ganglia networks. Eur. J. Neurosci. 28, 1437–1448 10.1111/j.1460-9568.2008.06422.x 18793321 PMC2756656

